# Assessment of compatibility of rhIGF-1/rhIGFBP-3 with neonatal intravenous medications

**DOI:** 10.1007/s12519-022-00610-9

**Published:** 2022-11-07

**Authors:** Nazila Salamat-Miller, Mark A. Turner, Amey Bandekar, Nitin Dixit, Emily Jochim, Barry Mangum, Christopher McPherson, Srini Tenjarla, Sukhjeet Singh, You Seok Hwang, Norman Barton

**Affiliations:** 1 Takeda, 200 Shire Way, Lexington, MA 02421 USA; 2 Institute of Translational Medicine, University of Liverpool, Liverpool, UK; 3 Paidion Research, Durham, NC USA; 4 Washington University, St. Louis, MO USA

**Keywords:** Chemical compatibility, Intravenous, Neonatal, Physical compatibility, rhIGF-1/rhIGFBP-3

## Abstract

**Background:**

Recombinant human (rh)IGF-1/IGFBP-3 protein complex, administered as a continuous intravenous infusion in preterm infants, is being studied for the prevention of complications of prematurity.

**Methods:**

We conducted in vitro studies to evaluate the physical and chemical compatibility of rhIGF-1/IGFBP-3 with medications routinely administered to preterm neonates. In vitro mixing of rhIGF-1/IGFBP-3 drug product with small-molecule test medications plus corresponding controls was performed. Physical compatibility was defined as no color change, precipitation, turbidity, gas evolution, no clinically relevant change in pH/osmolality or loss in medication content. Chemical compatibility of small molecules was assessed using liquid chromatography (e.g., reverse-phase HPLC and ion chromatography), with incompatibility defined as loss of concentration of ≥ 10%. A risk evaluation was conducted for each medication based on in vitro compatibility data and potential for chemical modification.

**Results:**

In vitro physical compatibility was established for 11/19 medications: caffeine citrate, fentanyl, fluconazole, gentamicin, insulin, intravenous fat emulsion, midazolam, morphine sulfate, custom-mixed parenteral nutrition solution (with/without electrolytes), parenteral nutrition solution + intravenous fat emulsion, and vancomycin (dosed from a 5 mg/mL solution), but not for 8/19 medications: amikacin, ampicillin, dopamine, dobutamine, furosemide, meropenem, norepinephrine, and penicillin G, largely owing to changes in pH after mixing. Small-molecule compatibility was unaffected post-mixing, with no loss of small-molecule content. For physically compatible medications, risk analyses confirmed low probability and severity of a risk event.

**Conclusion:**

Co-administration of rhIGF-1/rhIGFBP-3 drug product with various medications was assessed by in vitro studies using case-by-case risk analyses to determine the suitability of the products for co-administration.

**Supplementary Information:**

The online version contains supplementary material available at 10.1007/s12519-022-00610-9.

## Introduction

The rhIGF-1/rhIGFBP-3 drug product is the recombinant human (rh) version of the naturally occurring protein complex of insulin-like growth factor-1 (IGF-1) and its most abundant binding protein, insulin-like growth factor binding protein-3 (IGFBP-3). The product is currently under investigation for the prevention of complications of prematurity. Fetal IGF-1 concentration increases during the later stages of pregnancy and is important in the growth and development of the fetus [1]. For preterm infants, IGF-1 levels are lower during the first weeks of life than in fetuses of the same gestational age in utero [1]. In addition, lower levels of IGF-1 in extremely preterm infants have been associated with retinopathy of prematurity, bronchopulmonary dysplasia, and impaired neurodevelopment [1–7].

A 2019 phase 2 trial evaluated the use of rhIGF-1/rhIGFBP-3, formulated at 50 μg/mL isotonic acetate-based solution, at pH 5.5, with minor amounts of a surfactant, for the prevention of complications in extremely preterm infants (born at < 28 weeks’ gestational age) [8]. Although the trial’s primary endpoint of reduction in severity of retinopathy of prematurity was not met, substantial reductions were reported in the incidence of severe bronchopulmonary dysplasia and intraventricular hemorrhage. If the findings from the phase 2 trial are replicated in larger studies, there is a potential for rhIGF-1/rhIGFBP-3 to have an important impact on the clinical burden associated with preterm birth.

A previous pharmacokinetic modeling study predicted an rhIGF-1/rhIGFBP-3 dosing regimen of ≥ 250 μg/kg/24 h via continuous intravenous (IV) infusion to maintain serum IGF-1 levels in the physiological intrauterine range in extremely preterm infants [9]. However, IV access can be a challenge in preterm infants in the neonatal intensive care unit (NICU) because they typically require multiple IV therapies, including different classes of medications and parenteral nutrition (PN). Assurance of the compatibility of rhIGF-1/rhIGFBP-3 with other continuously infused medications is therefore of utmost importance. In the present study, a risk assessment strategy was designed to assess the feasibility and safety of co-administering rhIGF-1/rhIGFBP-3 with medications routinely administered IV in the NICU. This risk assessment strategy was based on the in vitro testing of physical and chemical compatibility and the theoretical potential for chemical incompatibility. Herein, we report the risk assessment methodology and findings for the medications evaluated.

## Methods

### Test medications

Test medications were selected on the basis of clinical priority (i.e., medications frequently administered to neonates), as identified by clinical experts and from investigative sites for the phase 2 trial [8]. The 19 medications included in this study were amikacin, ampicillin, caffeine citrate, dobutamine, dopamine, fentanyl citrate, fluconazole, furosemide, gentamicin, insulin, intravenous fat emulsion, meropenem, midazolam, morphine sulfate, norepinephrine bitartrate, penicillin G, custom-mixed PN solution (with and without electrolytes), PN solution + intravenous fat emulsion, and vancomycin (Supplementary Table 1 in the Supplementary Information). Most of the test medications were small-molecule drugs, whereas rhIGF-1/rhIGFBP-3 (Takeda, Lexington, MA, USA) is a recombinant protein.

### Risk assessment design and overview

**Fig. 1:**
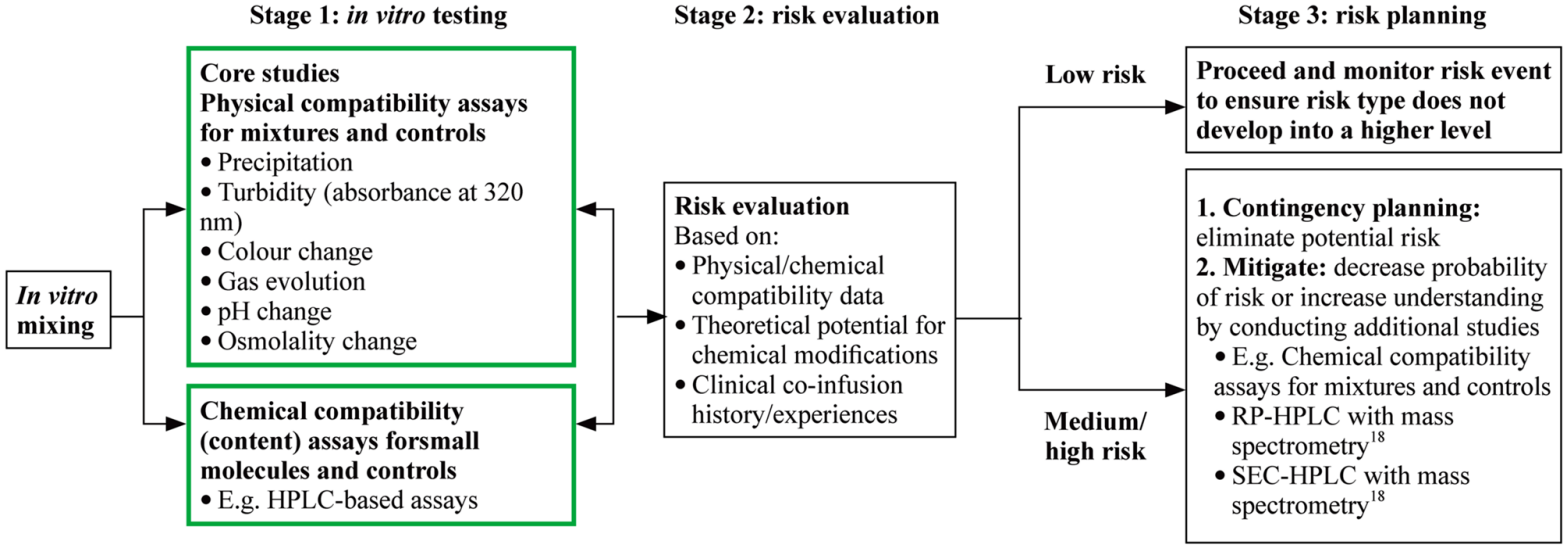
Risk assessment design. *RP-HPLC* reversed-phase high-performance liquid chromatography, *SEC-HPLC* size-exclusion high-performance liquid chromatography, *USP* United States Pharmacopeia. Green box denotes compendia and USP monograph assays

The risk assessment methodology was developed by a cross-functional team comprising clinicians, neonatal pharmacists, and representatives from the study sponsor’s product development departments for small molecules, biologics, clinical, and clinical operations. The risk assessment was composed of three consecutive stages (Fig. 1): (1) in vitro testing to determine the physical and chemical compatibility of rhIGF-1/rhIGFBP-3 with other medications (small molecules only); (2) a risk evaluation for each of the test medications, taking into account the known theoretical potential for chemical modifications, proximity to the isoelectric point of the protein when not in the mixture (based on pH value and probability of chemical modification), and the clinical co-infusion history (including co-administration with insulin [10], which shares a large homology with rhIGF-1); and (3) risk planning, based on an assessment of low, medium, or high risk of incompatibility.

### Mixing protocols

A mixing model was developed whereby rhIGF-1/rhIGFBP-3 and the test medication were mixed at one or more representative clinical doses. For all studies, the mixing calculations were performed with appropriate normalizations (time and neonates’ weights) to devise a volume-based scheme. For these calculations, a dose of 250 μg/kg/24 h (the dose used in the phase 2 trial) [8, 9] and at least two bracketing doses of the small-molecule medication were used (Table 1). For each study, multiple controls were designed to represent post-mixing concentrations and matrices of either rhIGF-1/rhIGFBP-3 or the test medication. The controls were created by diluting the rhIGF-1/rhlGFBP-3 drug product either with its own formulation buffer (to create a concentration control) or with the matrix of the small molecule to study the effect of change in the absence of any small molecule.

**Table 1:** rhIGF-1/rhIGFBP-3 drug product and small-molecule compatibility studies

Test medication^a^	Mixing dose for test medication	Preparation matrix per package insert	Time point for analysis (visual, pH, OD320)^b^	Notes
Amikacin sulfate	15 and 18 mg/kg/30 min	D5W	0, 60, 120 min; 24 h	Osmolality testing at 0, 60, 120 min, and 24 h
Ampicillin sodium	25 and 100 mg/kg/5 min	WFI followed by D5W	0, 30, 90 min; 3, 5, 8 h	
Caffeine citrate	20 mg/kg/20 min	N/A	0, 60, 120 min; 24 h	Osmolality testing at 0, 120 min, and 24 h
Dobutamine hydrochloride in dextrose	2, 10, and 25 µg/kg/min	N/A	0, 60, 90 min	
Dopamine hydrochloride	2, 5, 10, and 20 µg/kg/min	N/A	60 min	Osmolality testing was not part of the testing panel
Fentanyl citrate	0.5, 2, and 5 µg/kg/h	D5W	0, 60, 90 min	
Fluconazole	6 and 12 mg/kg/30 min	N/A	0, 60, 120 min; 24 h	Osmolality testing at 0, 60, 120 min, and 24 h
Furosemide	0.5, 1.0, and 2.0 mg/kg/5 min	N/A	0, 30, 60 min; 24 h	The observed turbidity was not associated with any furosemide content for up to 24 h, when assessed by RP-HPLC assay
Gentamicin	1, 3, and 5 mg/kg/30 min	N/A	0, 60, 120 min; 24 h	
Insulin	0.01, 0.05, and 0.1 units/kg/h	NS	0, 60, 90 min	
Intravenous fat emulsion	0.5 and 3 g/kg/day	N/A	0, 30, 60, 90 min	
Meropenem	10, 20, and 30 mg/kg/30 min	D5W	0, 15, 30, 60 min	
Midazolam hydrochloride	20 and 60 µg/kg/h	D5W	0, 60, 90 min	
Morphine sulfate	5, 10, and 50 µg/kg/h	D5W	0, 60, 90 min	
Norepinephrine bitartrate	0.05, 1.0, and 2.0 µg/kg/min	D5W	0, 60, 120 min; 24 h	
Penicillin G	25,000, 50,000, and 125,000 units/kg/30 min			
PN solution + intravenous fat emulsion	0.5 and 3 g/kg/day with 10 mL/h PN	N/A	0, 30, 60, 90 min	
PN solution (with and without electrolytes) – dextrose, 100 g/L	1, 4, and 10 mL/h	N/A	0, 30, 60 min	Osmolality testing was not part of the testing panel
Vancomycin hydrochloride	15 and 25 mg/kg/60 min	WFI	0, 60, 120 min; 24 h	Conducted as a worst-case study where a stock concentration of 50 mg/mL vancomycin was used for mixing studies
	15 and 25 mg/kg/24 h	WFI followed by D5W	0, 60, 90 min	A stock concentration of 5 mg/mL vancomycin was used for mixing studies

*D5W* 5% dextrose in water, *IGF-1* insulin-like growth factor-1, *IGFBP-3* insulin-like growth factor binding protein-3, *N/A* not applicable (no preparation/reconstitution/dilution was needed), *NS* normal saline, *OD320* optical density at 320 nm, *PN* parenteral nutrition, *rh* recombinant human, *RP-HPLC* reversed-phase high-performance liquid chromatography, *WFI,* water for injection

^a^The medications were prepared per each package insert; when applicable, each medication was diluted with the recommended diluents (0.9% normal saline, 5% dextrose and sterile water for injection)

^b^Osmolality was tested at 60, 90, and 120 min and at 24 h except where otherwise specified

Where applicable, the mixing duration was based on the calculated average infusion rate of rhIGF-1/rhIGFBP-3 (e.g., 250 μg/kg/24 h dose normalized for a 0.5 kg neonate and the target rhIGF-1/rhIGFBP-3 protein concentration of 50 μg/mL) with each test medication at the highest dose (normalized for the same weight) and an estimated volume for an umbilical catheter (Table 1). In all studies, periodic sampling of the mixture and control solutions was performed [11]. Additionally, longer mixing durations were considered as worst-case scenarios (e.g., interruptions could occur in clinical practice, extending the duration of the infusion) and to observe the continuation of any observed phenomena that occurred at the onset of mixing.

Two administration scenarios were assumed for the mixing duration calculations, where applicable. The first scenario was when each of the two medications was administrated via a pump, where the average of the two flow rates at the highest small-molecule dose was selected. In the second scenario, only rhIGF-1/rhIGFBP-3 was pumped, while the test medication was infused over a relatively short time (~ 15–30 min). For these scenarios and any other medications that were not pumped or infused, periodic observations and sampling occurred at selected time points (Table 1).

### Physical compatibility

Physical compatibility assays were compared for test samples and corresponding control solutions. In line with the existing literature [10, 12–16], we used the following methods, or modified versions thereof, to assess the physical compatibility of rhIGF-1/rhIGFBP-3 with the co-infused drugs: visual observation (United States Pharmacopeia [USP] < 790 >), optical density at 320 nm (USP < 851 > and < 857 >), pH measurements (USP < 791 >), and osmolality (USP < 785 >) at room temperature.

The aim of visual observation was to determine the presence of any precipitation, visible particulates, and flocculent matter, as well as any color change (compared with water) and/or gas formation, which are potential indicators of chemical modification(s).

All vials for physical compatibility testing were examined under the same lighting conditions: against a white and black background using both fluorescent light and Tyndall light (Spectralight III, Macbeth/X-Rite, Grand Rapids, MI, USA or MIH-DX, Bosch/Eisai Machinery, Waiblingen, Germany). Mixture and control samples were analyzed for appearance post mixing at specified time points (Table 1). Optical density measurements at 320 nm were carried out using an ultraviolet–visible spectrophotometer (SpectraMax M5, Molecular Devices, San Jose, CA, USA) for the detection of turbidity, an indicator of submicroscopic protein aggregation. Measurements were performed in triplicate using a 1 cm path-length quartz cuvette for each sample at each time point, and the average of these was recorded. pH values were recorded for each solution in triplicate using a calibrated pH meter (Model 215, Denver Instrument, Bohemia, NY, USA; Fischer Scientific Accumet XL150, Pittsburgh, PA, USA), and the average was reported. Osmolality changes post mixing at room temperature were recorded using a calibrated osmometer (Model 3250, Advanced Instruments, Norwood, MA, USA). Triplicate readings were ascertained, and the average was recorded.

Test medications were considered physically compatible with rhIGF-1/rhIGFBP-3 if there was no observed change in color, precipitation, turbidity, or gas evolution, or if there was no clinically relevant change in osmolality or pH.

Any change ±  ~ 0.3 pH of the mixtures from that of the rhIGF-1/rhIGFBP-3 drug product control (pH 5.5) was considered a change that could impact rhIGF-1/rhIGFBP-3 quality, in which case the small-molecule medication would be considered not compatible and would require further protein-specific data for evaluation. The considered range was based on the control and release specification of rhIGF-1/rhIGFBP-3 (5.5 ± 0.3) and a priori knowledge of the potential degradation and known stability of the rhIGF-1/rhIGFBP-3 drug product. The changes in osmolality values were considered using a less stringent criterion owing to existing clinical practices and in consideration of release specifications of rhIGF-1/rhIGFBP-3 (300 ± 30 mOsmol/kg) [17].

### Small-molecule chemical compatibility

The concentration of small-molecule test medications post mixing was assessed using either reversed-phase high-performance liquid chromatography (RP-HPLC), with ultraviolet (UV) detection, or ion chromatography with electrochemical detection at the last specified time point(s) (Table 1) (USP monographs or modified versions). For example, while a RP-HPLC–UV method was used for the detection of majority of molecules, for some, such as Amikacin and Gentamicin, a modified version of the USP ion chromatography assay with an electrochemical detection was used. For each medication, a qualification of the USP methods was conducted to ensure specificity, linearity, repeatability, and accuracy of the method. Example chromatograms for one small molecule (Gentamicin) are presented in the Supplementary information. Chemical incompatibility was considered to be a loss of the small-molecule content of ~ 10% or more over the defined testing period. Small-molecule analysis was not possible for PN or lipids owing to the complex nature of such mixtures.

### rhIGF-1/rhIGFBP-3 chemical compatibility

Sensitive mass spectrometry-based protein-specific methodologies have been developed by Takeda to assess the chemical compatibility of the rhIGF-1/rhIGFBP-3 drug product. The development of protein-specific methodologies is reported separately [18].

### Risk evaluation and risk planning

A comprehensive risk evaluation was completed for medications where in vitro (non)compatibility was indicated. (See *Risk assessment design and overview* in *Methods* for a description of the risk evaluation). A risk event was defined as “rhIGF-1/rhIGFBP-3 is not compatible with the co-infused drug over the duration and condition of the simulated mixing studies”.

The risk evaluation was performed for each co-infused test medication to determine the probability and severity of a risk occurrence. Probability was defined as the likelihood of an effect on safety, efficacy, or quality; severity was defined as the severity of the impact should the risk event occur (Table 2). On the basis of the level of probability and severity (low, medium, or high), a risk planning strategy was developed for each medication (Table 3). The cross-functional team of subject matter experts performed the final assessments and endorsed the clinical recommendations.

**Table 2:** Risk assessment definition

Description of risk	Description of risk event
Probability	What is the probability of the risk event occurring? Likelihood of an effect on safety, efficacy, or quality • High: high risk based on scientific rationale (e.g., pH changes over time for the mixing duration and conditions) • Medium: moderate probability of chemical modification (e.g., pH post mixing is outside of the demonstrated long-term pH range for stability, but all other observations are consistent with control samples) • Low: impact is not expected based on scientific rationale (e.g., mixture pH is within pH range acceptable for both rhIGF-1/rhIGFBP-3 and the co-infused drug)
Severity	What will be the severity of the impact should the risk event occur? • High: major effect on patient safety and therapeutic or biotherapeutic efficacy and quality, and effects on the co-administrated drug efficacy and quality as demonstrated by physical incompatibility (e.g., precipitation) • Medium: moderate effect of chemical compatibility; loss of potency (content for small-molecule drugs) observed for the in-use duration and condition • Low: no effect on the core testing based on biotherapeutic release specification (e.g., the drugs are physically compatible, with no observable loss of content)

*IGF-1* insulin-like growth factor-1, *IGFBP-3* insulin-like growth factor binding protein-3, *rh* recombinant human

**Table 3:** Risk prioritization grid with associated risk planning

Risk prioritization grid				
SEVERITY	High	High severity Low probability	High severity Medium probability	High severity High probability
	Medium	Medium severity Low probability	Medium severity Medium probability	Medium severity High probability
	Low	Low severity Low probability	Low severity Medium probability	Low severity High probability
		Low	Medium	High
	Probability			

Risk planning				
Avoid/contingency (H/H, H/M, M/H)	• Change plan to eliminate potential risk • Contingency plan must be developed and in place ○ To be implemented when event occurs or at trigger point • Define trigger points for evaluation and ensure appropriate action is taken (e.g., activate contingency plan) • Example: avoid administrating the incompatible product. Communicate with clinical site on the risk of co-infusion. Switch to a compatible product			
Avoid/mitigate (M/M, L/H, H/L)	• Change plan to eliminate potential risk or plan how to minimize the impact (e.g., separate line for infusion and avoid co-infusion) • Define/set trigger points and take appropriate action where necessary ○ Examine each identified risk area ○ Isolate the cause ○ Develop response • Example: Avoid administrating the incompatible product. Use a separate line for infusion			
Passive acceptance (M/L, L/M, L/L)	• Accept and monitor risk to ensure risk type does not develop into a higher level • Example: Proceed with administration after collecting the initial physical data, augmented by a cross-functional risk-based assessment to support the co-administration			

*H* high, *L* low, *M* medium

## Results

### In vitro physical compatibility

Of the 19 medications tested, physical compatibility was established for rhIGF-1/rhIGFBP-3 with caffeine citrate, fentanyl, fluconazole, gentamicin, insulin, intravenous fat emulsion, midazolam, morphine sulfate, PN solution + intravenous fat emulsion, PN solution (with and without electrolytes), and vancomycin (when dosed from a 5 mg/mL solution) (Table 4). The following medications were considered incompatible with rhIGF-1: amikacin, ampicillin, dobutamine, dopamine, furosemide, meropenem, norepinephrine, penicillin G, and vancomycin (when dosed from a 50 mg/mL solution).

**Table 4:** Physical and small-molecule compatibility of the rhIGF-1/rhIGFBP-3 drug product and small-molecule test medications

Test medication	Physical compatibility	Chemical compatibility for small-molecule content (RP-HPLC)	Notes
Amikacin sulfate	No	Yes	Mixtures demonstrated a decrease in pH of ~ 0.6 units compared with controls; difference of ~ 12% in the osmolality of mixture samples compared with controls
Ampicillin sodium	No	Yes	Observed change in pH of rhIGF-1/rhIGFBP-3 post mixing beyond the pH range that maintains stability; mixture pH was too close to the isoelectric point of rhIGF-1/rhIGFBP-3
Caffeine citrate	Yes^a^	Yes	pH of the mixed rhIGF-1/rhIGFBP-3 and caffeine samples at two different doses demonstrated ~ 1.0 unit difference compared with the control; osmolality of mixture samples was ~ 40% lower compared with the rhIGF-1/rhIGFBP-3 sample (attributable to differences in formulation matrices between rhIGF-1/rhIGFBP-3 and caffeine citrate); however, significant volume of protein-specific studies confirmed the compatibility (see footnote)
Dobutamine hydrochloride	No	Yes	Although the medication demonstrated physical compatibility (no precipitation, no visible particles, no turbidity, no color change, and no gas evolution), on the basis of gained knowledge using protein-specific assays in the presence of sodium (meta)bisulfite^b^ the medication is considered incompatible
Dopamine hydrochloride	No	Yes	A dopamine dose-dependent physical change (pH change) was observed, not concurrently with any other changes (no visible particles, no turbidity, no color change, and no gas evolution). Additionally, the protein-specific data demonstrated an impact on the rhIGF-1/rhIGFBP-3 drug product in the presence of sodium (meta)bisulfite^b^
Fentanyl	Yes	Yes	An osmolality decrease of ~ 19% was observed for fentanyl at the highest dose of 5 µg/kg/h, as compared with the rhIGF-1/rhIGFBP-3 drug product (attributed to dilution of rhIGF-1/rhIGFBP-3 formulation buffer), but the decrease was not considered clinically significant
Fluconazole	Yes	Yes	
Furosemide	No	Yes	Cloudiness observed within ~ 30 min of mixing
Gentamicin	Yes	Yes	Osmolality decrease of ~ 74% was observed with the highest dose of 5 mg/kg/30 min but was not considered clinically significant because the low osmolality was observed for the gentamicin medication itself used at this clinically relevant dose
Insulin	Yes	Yes	Insulin monograph assay faced low recovery at low insulin doses; therefore, higher insulin concentrations were tested to obtain recovery and observe any incompatibility trends
Intravenous fat emulsion	Yes	N/A	No disruption of the emulsion, phase separation, color change, or lipid flocculation was observed upon mixing of the drug product and the fat emulsion (Intralipid® 20%) pH increase of 0.2 observed after mixing rhIGF-1/rhIGFBP-3 drug product with intravenous fat emulsion (up to 90 min), due to the approximate pH value of 8.0 for the intravenous fat emulsion; the increase in pH of the mixture is within the drug stability pH specification so was not considered clinically significant
Meropenem	No	Yes	Observed pH change of ~ 2.4 post mixing, beyond the pH range that maintains the stability of rhIGF-1/rhIGFBP-3
Midazolam	Yes	Yes	
Morphine sulfate	Yes	Yes	
Norepinephrine bitartrate	Yes (only the lowest dose)^c^	Yes	Only the lowest dose was physically compatible with respect to the pH change; however, the small-molecule medication is formulated with sodium (meta)bisulfite; therefore, we do not consider this drug to be compatible when formulated as such
Penicillin G	No	Yes	Further protein-specific investigations are ongoing to clarify the impact of the observed pH shift (up to 1.3 pH units for the highest penicillin G dose of 125,000 Units/kg/30 min) on rhIGF-1/rhIGFBP-3
PN solution + intravenous fat emulsion	Yes	N/A	No pH change, disruption of the emulsion, phase separation, color change, or lipid flocculation was observed upon mixing of the drug product, PN (a high dose of 10 mL/h), and two doses of the fat emulsion (Intralipid® 20%)
PN solution (with and without electrolytes)	Yes	N/A	No precipitation, particulate formation, or mixture turbidity (as assessed visually and by optical density at 320 nm) was observed No change in the pH of the final mixture of PN and drug product was observed
Vancomycin hydrochloride	Yes^c^	Yes	Compatible only when dosed from a stock vancomycin concentration of 5 mg/mL; if infused at higher concentration (50 mg/mL), the change in pH is significant owing to the presence of hydrochloric acid in vancomycin’s formulation

*IGF-1* insulin-like growth factor-1, *IGFBP-3* insulin-like growth factor binding protein-3, *N/A* not applicable, *PN* parenteral nutrition, *rh* recombinant human, *RP-HPLC* reversed-phase high-performance liquid chromatography

^a^To date, on the basis of these studies and protein-specific analyses, this medication has been removed from the incompatible list of medications. The protein-specific analyses demonstrated that the observed pH and osmolality changes did not cause fragmentation, oxidation, aggregation, adduct formation, and generation of any free rhIGF-1 or rhlGFBP-3 submolecular units

^b^ “Development of Protein-Specific Analytical Methodologies to Evaluate Compatibility of Recombinant Human (rh)IGF-1/rhIGFBP-3 with Intravenous Medications Co-Administered to Neonates”

^c^Compatible based on the physical data; however, the final compatibility decision is informed by other studies [18]

### Small-molecule chemical compatibility

Small-molecule compatibility was not affected post mixing for the medications tested. No loss of small-molecule content was observed for any of the medications tested in the mixture and corresponding controls (Table 1).

### Risk evaluation and risk planning

Risk evaluations were completed for all small-molecule test medications (except furosemide, where the studied mixture became turbid within ~ 30 min and clearly indicated incompatibility with the rhIGF-1/rhIGFBP-3 drug product). Where in vitro physical compatibility was confirmed, the subsequent risk evaluations confirmed a low probability and severity of an event within the context of in-use conditions. The risk of interaction or chemical modification, based on pH values, also was considered to be low for those medications showing in vitro compatibility.

Given the structural similarity between insulin and IGF-1 [19], medication compatibility with insulin was given significant consideration when evaluating rhIGF-1/rhIGFBP-3 compatibility with tested medications. For example, compatibility with insulin has been established for midazolam and vancomycin [10], suggesting a low risk of incompatibility with rhIGF-1/rhIGFBP-3; however, particular attention was given to the small-molecule concentrations that were assessed in regard to compatibility with insulin. No compatibility data are available to date for insulin with fentanyl or fluconazole; however, on the basis of theoretical evaluation and the solution pH, a reaction is not expected.

Among the drugs that were observed to be incompatible with rhIGF-1/rhIGFBP-3 in the in vitro testing studies, the risk was classified as medium/high and appropriate actions were recommended. An example of a risk assessment, for morphine, is provided in Supplementary Table 2 in the Supplementary Information.

## Discussion

In this study, in vitro testing indicated the physical compatibility of the rhIGF-1/rhIGFBP-3 drug product with 11/19 medications and nutritional therapies under the conditions and doses tested. For medications showing in vitro compatibility, the risk evaluation confirmed a low probability and severity of risk for incompatibility. Physical compatibility was not established with 8/19 medications. For drugs identified as incompatible, infusions would need to be redistributed to optimize available IV lines.

Historically, there has been a lack of compatibility data available for drugs administered to preterm infants. A review of neonatal drug studies found that no documentation on compatibility was available for almost 60% of IV drug-drug infusions, and for 34% of IV drug-nutrition co-infusions administered in the NICU [20].

As might be expected, there is a lack of comprehensive drug testing in neonates. One US study reported that only 35% of medications administered to neonates were approved by the US Food and Drug Administration for use in infants [21], and an Italian study found that 44% of medications were prescribed off-label in the preterm neonatal population [22]. Such findings have potential implications for both treatment efficacy and safety in infants. The majority of compatibility studies are performed for small molecules co-administered with small molecules; literature was identified for compatibility testing for only two biologics, insulin and vasopressin [12, 14], and standard biologic-specific testing methods have not been established.

In this study we investigated the physical compatibility of the rhIGF-1/rhIGFBP-3 drug product when mixed with frequently administered medications. To generate a complete picture of compatibility, we evaluated chemical compatibility at the level of the small-molecule content as part of a separate study on the protein content/chemical modification [18]. It should be noted that the main challenge with the protein-specific methodologies is the extremely low protein concentration of rhIGF-1/rhIGFBP-3 post mixing with each small molecule. The low concentrations require highly specific and robust analytical methods.

rhIGF-1/rhIGFBP-3 is being investigated for the prevention of complications of prematurity, with an ongoing comprehensive clinical trials program to evaluate the safety and efficacy of the drug. The present work is being conducted as part of this program, to systematically evaluate and build a comprehensive body of data on the compatibility of rhIGF-1/rhIGFBP-3 with commonly administered intravenous drugs. Our goal is eventually to evaluate a large panel of medications and to build up a comprehensive body of data to aid clinicians’ decision-making regarding the co-infusion of rhIGF-1/rhIGFBP-3. Drug compatibility testing studies should be conducted as early as feasible in the investigational phase of a neonatal drug to allow sufficient time for study findings to inform clinical trials as well as the eventual adoption of the drug in clinical practice.

An inherent limitation of the current study is the fact that the results are based on in vitro mixing under specific controlled conditions, and the risk evaluation is theoretical. Careful monitoring will be required in the clinical setting. The risk assessment will evolve when more protein-specific data become available. For example, recent protein-specific analyses demonstrated that the presence of the excipient sodium (meta)bisulfite is damaging to rhIGF-1/rhIGFBP-3 owing to its oxidation. Therefore, although no physical (pH) change was observed for the dobutamine and norepinephrine mixtures, these medications have been removed from the compatible list because they are formulated with sodium (meta)bisulfite. Additionally, the presence of (meta)bisulfite will be added to the risk analysis strategy.

In conclusion, the administration of rhIGF-1/rhIGFBP-3 with various medications will be facilitated by conducting selected in vitro studies and case-by-case risk assessments. This study adds a novel approach to the compatibility testing of drugs utilized in the NICU and may set a benchmark for future studies. Early contributions from clinicians and cross-functional disciplines will be essential to the process.

## Supplementary Information

Below is the link to the electronic supplementary material.

Supplementary file1 (DOCX 592 KB)

## Data Availability

The datasets, including the redacted study protocol, supporting the results reported in this article will be made available within three months from initial request to researchers who provide a methodologically sound proposal.
