# Supplementary material for: Assessment of compatibility of rhIGF-1/rhIGFBP-3 with neonatal intravenous medications
Source: World J Pediatr. 2022 Nov 7;19(1):58–67. doi: 10.1007/s12519-022-00610-9 (PMC9832074; doi:10.1007/s12519-022-00610-9)
Supplement: Supplementary file 1 — Supplementary file1 (DOCX 592 KB) [file 12519_2022_610_MOESM1_ESM.docx]

**Assessment of compatibility of rhIGF-1/rhIGFBP-3 with neonatal intravenous medications**

**Supplementary information**

**Supplementary Table 1** Medications tested in the compatibility study

| **Medication** | **Manufacturer** |
| --- | --- |
| Amikacin sulfate | Teva Parenteral Medicines, Irvine, CA, USA |
| Ampicillin | Sandoz Inc., Princeton, NJ, USA |
| Caffeine citrate | Sagent Pharmaceuticals, Schaumburg, IL, USA |
| Dobutamine HCl | Baxter, Deerfield, IL, USA |
| Dopamine HCl | Hospira Inc., Lake Forest, IL, USA |
| Fentanyl citrate | Hospira Inc., Lake Forest, IL, USA |
| Fluconazole | Sagent Pharmaceuticals, Schaumburg, IL, USA |
| Furosemide | Hospira Inc., Lake Forest, IL, USA |
| Gentamicin | APP Pharmaceuticals, Los Angeles, CA, USA |
| Insulin (Novalin R) | Novo Nordisk, Bagsværd, Denmark |
| Intravenous fat emulsion (Intralipid^®^ 20%) | Fresenius Kabi, Bad Homburg, Germany |
| Meropenem | Hospira Inc., Lake Forest, IL, USA |
| Midazolam HCl | Akorn, Lake Forest, IL, USA |
| Morphine sulfate | Hospira Inc., Lake Forest, IL, USA |
| Norepinephrine bitartrate (Levophed) | Hospira Inc., Lake Forest, IL, USA |
| Penicillin G potassium | Sandoz Inc., Princeton, NJ, USA |
| PN solution (with and without electrolytes)^a^ | Solutions were prepared by Tufts University, Medford, MA, USA |
| PN solution + Intravenous fat emulsion (Intralipid 20%)^a^ | Solutions were prepared by Tufts University and mixed in house |
| Vancomycin HCl | Hospira Inc.; Mylan, Canonsburg, PA, USA |

*PN* parenteral nutrition

^a^Solutions were studied within 24 h of preparation.

**Supplementary methods: small molecule chemical compatibility**

For each medication, a qualification of the USP methods (or its modified version) was conducted to ensure specificity, linearity, repeatability, and accuracy of the method. Gentamicin is presented in this section as an example. Amino sugar-driven antibiotics such as Gentamicin and its related substances were analyzed by ion chromatography with electro chemical detection. The USP monograph for the Content of Gentamicin in Gentamicin Sulfate was modified to quantitate the assay (concentration) of Gentamicin Sulfate. Several modifications were made to the USP monograph to obtain these results. Samples were quantitated using a n=5 standard calibration with a nominal standard concentration of 0.2 mg/mL Gentamicin Sulfate. The combined area (group) of all Gentamicin peaks was used to quantitate the amount of Gentamicin Sulfate (mg/mL) in samples. Mixture and control samples were then analyzed per the modified method to support the study. Mixture samples were diluted in mobile phase to obtain a suitable working concentration for all samples. **Supplementary** **Table 2** summarizes the analysis parameter for the presented example. **Supplementary** **Figures 1-6** show the representative chromatograms for Gentamicin.

**Supplementary Table 2** Methods: Chromatographic Conditions

| HPLC / Ion Chromatography System: | Dionex ICS-3000 | | |
| --- | --- | --- | --- |
| Detection Mode: | Integrated Amperometry | | |
| Mobile Phase: | TFA (trifluoroacetic acid) with pentafluoropropanoic acid, 50% Sodium Hydroxide (carbonate free), and acetonitrile, pH 2.6 | | |
| Post-column Reagent: | 20 g/L Sodium Hydroxide (Carbonate-free) | | |
| Flow Rate: | 1. mL/minute Mobile Phase (Pump 1)   0.3 mL/minute Post-column Reagent (Pump 2) | | |
| Column: | YMC Pack Pro C18 4.6 x 250 mm, 5 uM | | |
| Column Temperature: | 35 °C | | |
| Autosampler Temperature: | Ambient (not controlled) | | |
| Injector Volume: | 20 µL | | |
| Offset Level: | 10% | | |
| Range: | 300 uC | | |
| Run Time: | 50 minutes | | |
| Waveform: | Time (seconds) | Potential (V) | Integration |
|  | 0.00 | 0.05 |  |
|  | 0.10 | 0.05 | On |
|  | 0.40 | 0.05 | Off |
|  | 0.41 | 0.75 |  |
|  | 0.55 | 0.75 |  |
|  | 0.56 | -0.15 |  |
|  | 1.00 | -0.15 |  |

Supplementary Figure 1 Example of Standard A Solution Chromatogram


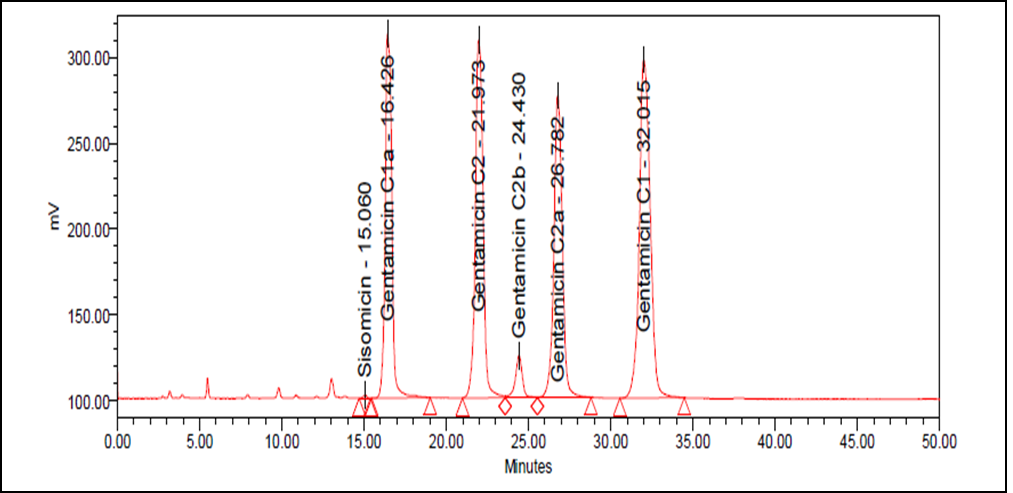


Supplementary Figure 2 Overlay Chromatogram of (rhIGF-1/rhIGFBP-3) Formulation Buffer and Gentamicin Solution (in Water for Injection) Standard Solution


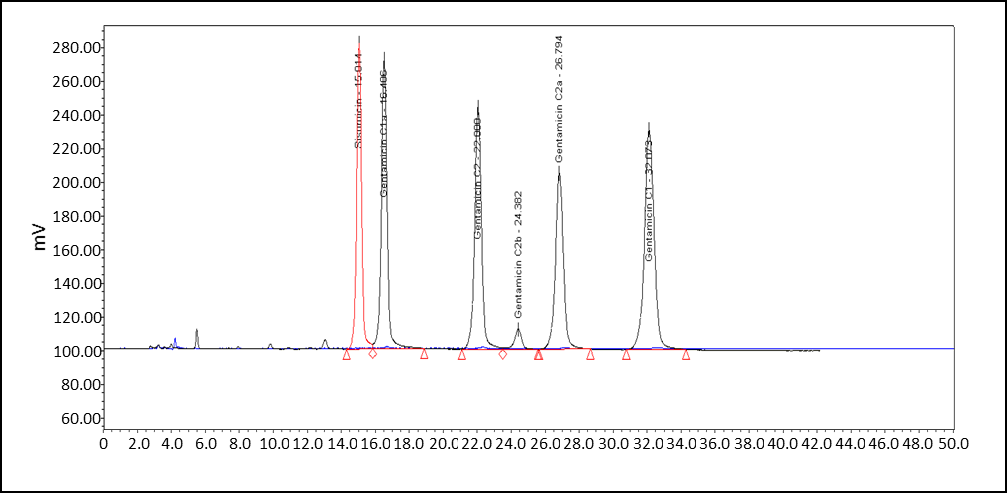


Supplementary Figure 3 Overlay Chromatogram of rhIGF-1/rhIGFBP-3 Drug Product and Gentamicin Solution (in Water for Injection) Standard Solution


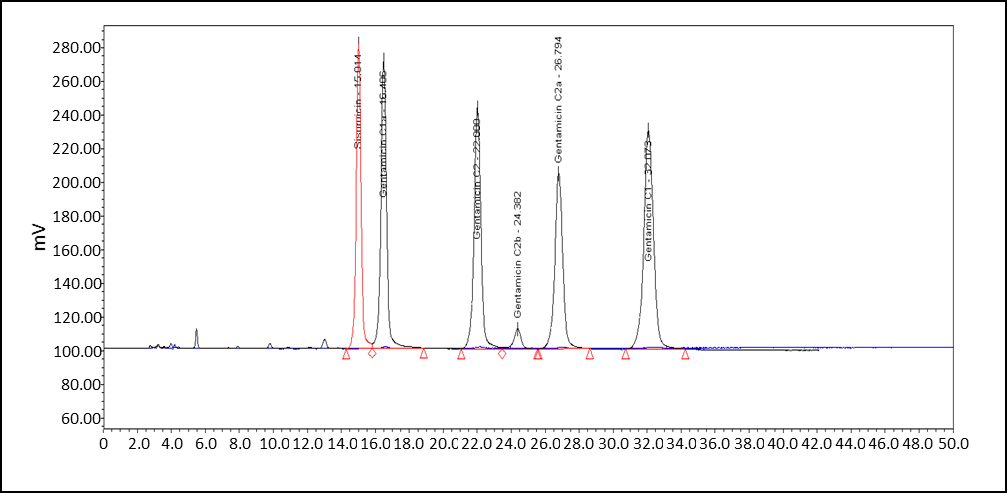


**Supplementary** **Figure 4** Example of rhIGF-1/rhIGFBP-3 Drug Product Control

**
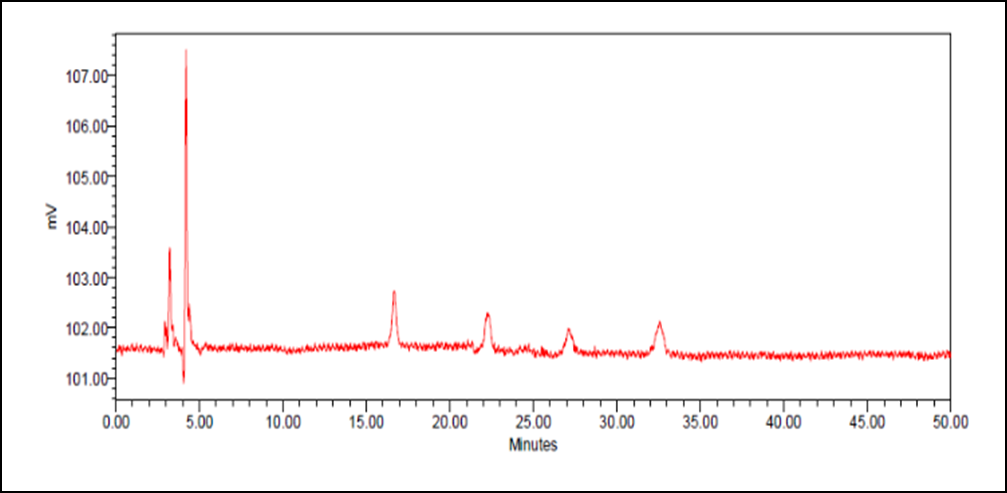
**

**Supplementary** **Figure 5** Example Gentamicin (at 1 mg/kg/30 min dose) Chromatogram post Mixing with rhIGF-1/rhIGFBP-3 Drug Product

**
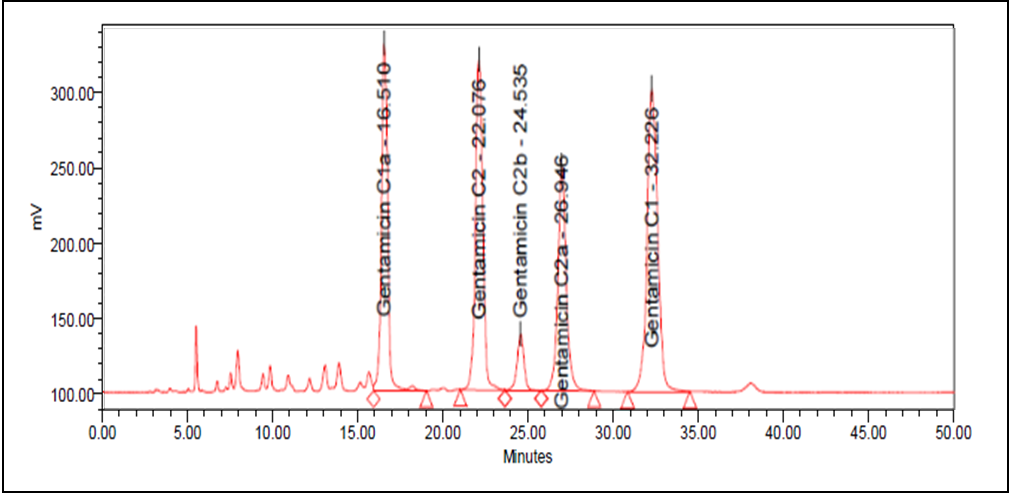
**

**Supplementary** **Figure 6** Example Gentamicin (at 5 mg/kg/30 min dose) Chromatogram post Mixing with rhIGF-1/rhIGFBP-3 Drug Product


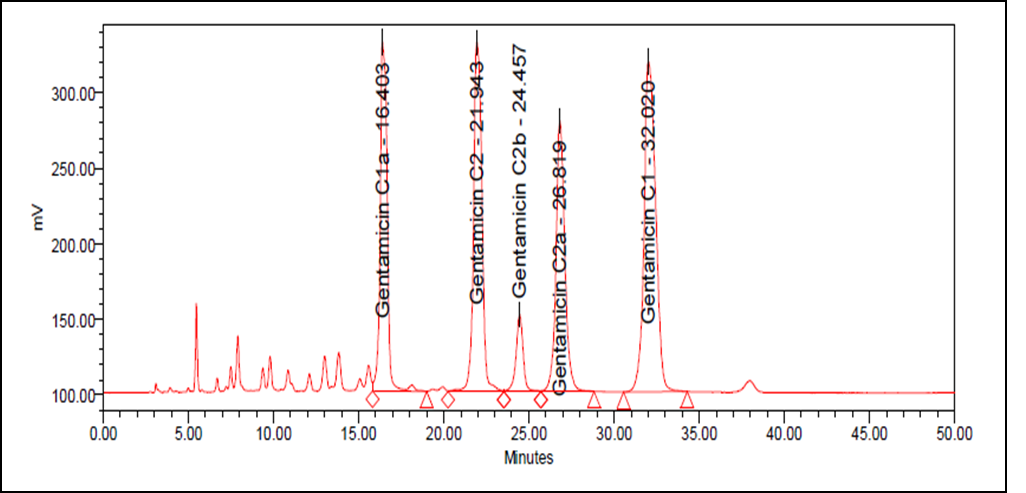


**Supplementary Table 3** Risk evaluation for morphine

| **Description of risk** | **Probability/severity**  **risk evaluation****^a^** | **Explanation of risk evaluation rating** | **Strategy^b^** |
| --- | --- | --- | --- |
| Morphine solution is not compatible with rhIGF-1/ rhIGFBP-3 for the in-use duration and conditions | Low probability  Low severity | - Morphine and rhIGF-1/rhIGFBP-3 are physically compatible based on drug-drug compatibility study data for in-use conditions (low severity) - There is no loss of morphine content in the presence of rhIGF-1/rhIGFBP-3 drug product on the basis of drug-drug compatibility study data for in-use conditions (low severity) - Osmolality range of mixture is acceptable for infusion drugs (low probability, low severity) - pH range of mixture is acceptable for both rhIGF-1/rhIGFBP-3 (5.5 ± 0.3) and morphine (2.5–6.5; note: reference point for nondiluted samples only)   - No physical incompatibility is observed or expected (low severity)   - Low probability for chemical incompatibility from solution pH (low probability) - Risk of interaction between morphine and rhIGF-1/rhIGFBP-3 is low within the context of in-use conditions; chemical modification of small molecules and protein is expected to occur over a longer duration compared with the in-use duration of ~1 h, calculated on the basis of in-use conditions; probability of degradation is expected to be minimal (low probability) - Morphine and insulin are compatible [[10](#_ENREF_10)]; however, there are currently no data for morphine and rhIGF-1/rhIGFBP-3 by protein-specific methodology | - Passively accept and monitor   - Clinical recommendation of co-infusion; additional chemical compatibility characterization and clinical monitoring should also be considered |

*IGF-1* insulin‑like growth factor-1, *IGFBP‑3* insulin‑like growth factor binding protein‑3

**^a^**Options: High, medium, or low.
**^b^**Options: Mitigation plan, contingency plan, or passively accept and monitor.
